# Genome-Wide Characterization of Wholly Disordered Proteins in *Arabidopsis*

**DOI:** 10.3390/ijms26031117

**Published:** 2025-01-28

**Authors:** Wenfen Long, Liang Zhao, Huimin Yang, Xinyi Yang, Yulong Bai, Xiuhua Xue, Doudou Wang, Shengcheng Han

**Affiliations:** 1 Beijing Key Laboratory of Gene Resources and Molecular Development, College of Life Sciences, Beijing Normal University, Beijing 100875, China; 202321200015@mail.bnu.edu.cn (W.L.); zhaoliang20181012@163.com (L.Z.); yhm_2024@163.com (H.Y.); yangxinyi2024@sibcb.ac.cn (X.Y.); byl1605164599@163.com (Y.B.); xiuhuaxue@bnu.edu.cn (X.X.); 2 Academy of Plateau Science and Sustainability of the People’s Government of Qinghai Province & Beijing Normal University, Qinghai Normal University, Xining 810008, China

**Keywords:** WDPs, physicochemical properties, expression, PPI, liquid-liquid phase separation, *Arabidopsis*

## Abstract

Intrinsically disordered proteins (IDPs) include two types of proteins: partial disordered regions (IDRs) and wholly disordered proteins (WDPs). Extensive studies focused on the proteins with IDRs, but less is known about WDPs because of their difficult-to-form folded tertiary structure. In this study, we developed a bioinformatics method for screening more than 50 amino acids in the genome level and found a total of 27 categories, including 56 WDPs, in *Arabidopsis*. After comparing with 56 randomly selected structural proteins, we found that WDPs possessed a more wide range of theoretical isoelectric point (PI), a more negative of Grand Average of Hydropathicity (GRAVY), a higher value of Instability Index (II), and lower values of Aliphatic Index (AI). In addition, by calculating the FCR (fraction of charged residue) and NCPR (net charge per residue) values of each WDP, we found 20 WDPs in R1 (FCR < 0.25 and NCPR < 0.25) group, 15 in R2 (0.25 ≤ FCR ≤ 0.35 and NCPR ≤ 0.35), 19 in R3 (FCR > 0.35 and NCPR ≤ 0.35), and two in R4 (FCR > 0.35 and NCPR > 0.35). Moreover, the gene expression and protein-protein interaction (PPI) network analysis showed that WDPs perform different biological functions. We also showed that two WDPs, SIS (Salt Induced Serine rich) and RAB18 (a dehydrin family protein), undergo the in vitro liquid-liquid phase separation (LLPS). Therefore, our results provide insight into understanding the biochemical characters and biological functions of WDPs in plants.

## 1. Introduction

The lock-and-key hypothesis proposed by Emil Fischer highlights the requirement for complementary and matching structures of given substrates to fit to an enzyme [1]. This model solidifies the conception of structure-function continuum. With the development of X-ray crystal diffraction, NMR, and other experimental techniques, researchers have realized that the structures of proteins are closely related to their biological functions. However, not all proteins have a highly specific three-dimensional structure when they function in biological process [2,3,4,5,6]. Increasing evidence has suggested that disordered conformation and flexible structure hold key positions in the function of proteins [7]. The above findings challenge the traditional structure-function paradigm [8]. Under physiological conditions, intrinsically disordered proteins (IDPs) are unable to form specific secondary or 3D structures but nevertheless participate in various processes. IDPs are divided into two types: proteins with partially disordered regions (IDRs), which confer the conformational flexibility to IDPs, and wholly disordered proteins (WDPs) [9]. When combined with different molecules, IDPs shift their binding modes and experience a transition from disorder to order, so that they ensemble specific conformations to function [10,11]. The dynamic transformation of conformation enables IDPs to interact with proteins or nucleic acids, trigger liquid-liquid phase separation (LLPS), and regulate membraneless organelles, etc. [12,13].

IDPs are widely distributed throughout the biological kingdom and are particularly prevalent in complex eukaryotes, where they account for about 40% of all proteins [14,15]. Previously, studies showed that IDPs often form biomolecular polymers via LLPS, dependent on their IDRs [16]. In mammals, aberrant IDP expression has been implicated in many diseases, such as cancers, cardio-cerebrovascular, and neurodegenerative diseases [17,18,19]. For example, as one of the identified IDPs, Tau’s aggregation contributes to Alzheimer’s disease (AD) [20], and phosphorylation modification promotes the phase separation of Tau and accelerates the formation of amyloid protein [21]. In plants, IDPs affect transcriptional regulation and post-translational modification, and participate in the process of signal transduction, disease resistance, and stress response [22,23]. Previous studies have found that plant viruses hijack the cell phase separation mechanism and affect the host’s transcription and translation processes [24]. The Late Embryogenesis Abundant (LEA) protein gene family is a group of disordered proteins that have been widely studied in plants including *Solanum lycopersicum*, *Prunus mume,* and *Arabidopsis thaliana* [25,26,27]. When plants are exposed to extreme conditions, LEA is highly expressed and even ensures plant survival through complete loss of water [25,28,29]. When transformed into other plants, LEAs improve the stress resistance of the transgenic plants. For example, overexpression of Barley *HVA1* gene in wheat and *Oryza sativa* enhance their tolerance to water shortage and significantly increase their water utilization [30,31]. In summary, IDPs are essential for a wide range of physiological processes in complex biological systems.

In recent years, a variety of computational prediction methods have been developed to characterize IDPs, based on different training purposes and different data sets [32,33]. DisProt has become the gold standard in IDP/IDR annotation, and the number of experimentally validated IDPs in the DisProt has increased substantially [34,35]. DisProt 9.5 contains 2896 entries of IDPs, including relevant data for *Arabidopsis*, mice, yeast and other species. In *Arabidopsis*, 100 IDPs are verified. Among them, 12 members are completely disordered, which correspond to the LEA family (including dehydrin), the Calvin cycle protein CP12-2, and the protein COLD-REGULATED 15A [36,37]. However, the classification of IDPs is chaotic. In previous studies, proteins with partial disordered regions have been studied well for their ordered regions possessing obvious physiological roles, instead, the study of WDPs were challenged by its structural characterization [9,38]. The properties of WDPs and the distinctions in their expression patterns under different circumstances remain poorly understood. Moreover, the effect of whole-chain disorder on intermolecular interaction and the specific affinity of their binding sites, as well as bonds with other molecules, need to be further elucidated.

Here, we present a clear definition of wholly disordered proteins (WDPs) in *Arabidopsis*, which represent proteins with nearly 100% of highly disorder. A set of criteria was developed based on the *Arabidopsis* Proteome and predicted structures from AlphaFold and MobiDB, which were used to screen out 56 WDPs with disorder degrees of 90% to 100%. A comprehensive evaluation was conducted to assess their physicochemical properties, evolutionary characteristics and expression patterns under four different abiotic stress treatments. To further comprehend the potential biological roles of selected WDPs, we proceeded to predict their interactions with others, and these WDPs underwent GO analysis in the process. In vitro phase separation assay shows that two WDPs, *SIS* and *RAB18*, are capable of form LLPS in vitro, which provides insights for future research on these proteins in plants.

## 2. Results

### 2.1. Identifing WDPs from the Uniprot Proteme

In accordance with the aforementioned criteria, which stipulate those disordered regions occupy 90~100% of its total length and structural information provided by AlphaFold and MobiDB, a comprehensive search of the Uniprot was conducted to find all entries related to WDPs. The database of such “wholly disordered” complexes contained 27 categories of 56 WDP members in *Arabidopsis* (Table 1), accounting for approximately 0.14% of all proteins.

**Figure 1:**
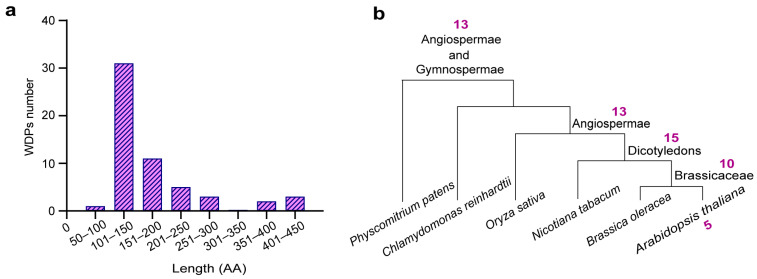
Wholly disordered protein (WDPs) in *Arabidopsis*. (**a**) Length distribution of WDPs in *Arabidopsis*. (**b**) Distribution of homologous WDPs in the plants. The counts denote the number of homologous WDPs.

We firstly found that a small part of WDPs can be assigned to known protein families, including PKS (Protein PHYTOCHROME KINASE SUBSTRATE), LEA (Late embryogenesis abundant protein), SPIRAL1 (Protein SPIRAL1-like), and SOFL (Protein SOB FIVE-LIKE). The rest of the WDPs could not to be classified into any known proteins. Instead, these proteins were grouped together based on their great similarity and shared conserved areas (Figure S1). Possibly, these homologous WDPs, with similar amino acid substitutions, may adopt analogous folding patterns and perform the same or similar biochemical functions. The lengths of WDPs varied from 99 (*SP1L5*) to 442 amino acids (*PKS2*), with a prevalent distribution in the range of 100~200 amino acids (Figure 1a). Chain lengths and sequence features represent important factors determining the compaction degree of IDPs, which might correlate with the propensity to phase separation [39,40]. Such short lengths of these WDPs may lead to unsuccessful amplification of weak intramolecular interactions, accompanied by increasing probability of functional misfolding.

To understand the evolutionary relatedness of WDPs in plant lineages, we conducted a synteny analysis of the WDPs among *Arabidopsis thaliana*, *Brassica oleracea*, *Nicotiana tabacum*, *Oryza sativa*, *Physcomitrium patens,* and *Chlamydomonas reinhardtii*. The results were classified and displayed based on the presence of homologous protein clusters in various plants (Figure 1b, Table S1). Figure 1b presented that there were five specific WDPs (*F4I179*, *Q9M3G8*, *EFC*, *A0A1P8ASG6*, and *CCaP1*) in *Arabidopsis Thaliana*, 10 WDPs in Brassicaceae, 15 WDPs in *Dicotyledons*, and 13 WDPs in *Angiospermae*. It is worth noting that *Angiospermae* and *Gymnospermae* shared 13 common WDPs. WDPs have several sequence repeats and generally evolve at a comparatively faster rate, which underlines that the repetitive segments increase with organism complexity and may be shaped by intense evolutionary activities [41,42]. Species-specific WDPs may emerge early and subsequently be retained over the process of evolution and species differentiation.

### 2.2. Comparison of Physicochemical Properties Between WDPs and Structural Proteins

To gain deeper insight into specific features of WDPs and structural proteins, these WDPs were systematically compared with 56 ordered proteins with 100~600 amino acids (Table S2), which were randomly selected from UniProt Proteome. We first assessed four chosen features, the Theoretical pI (PI), Grand Average of Hydropathicity (GRAVY), Instability Index (II), and Aliphatic Index (AI).

**Figure 2:**
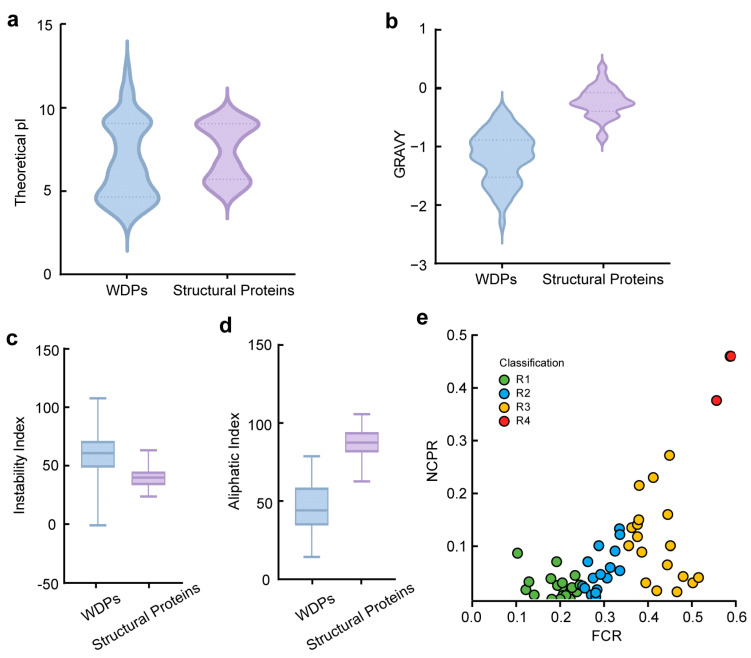
Comparison of physicochemical properties between the WDPs and 56 structural proteins. (**a**) Comparison of average PI. (**b**) Comparison of total average hydrophilicity (GRAVY). (**c**) Comparison of Instability Index (II). (**d**) Comparison of Aliphatic Index. (**e**) Classification of *Arabidopsis* WDPs based on FCR and NCPR values. R1: FCR < 0.25 and NCPR < 0.25, R2: 0.25 ≤ FCR ≤ 0.35 and 0.25 ≤ NCPR ≤ 0.35, R3: FCR > 0.35 and NCPR ≤ 0.30, R4 and R5: FCR > 0.35 & NCPR > 0.30.

The majority of WDPs were found to be weakly acidic or neutral, and their distribution of PI was more polarized (Figure 2a). The average PI values of WDPs were 6.770, with a mean PI of 6.380. Structural proteins exhibited a smaller PI variation range, from 5 to 7, with mean and average PI values of 7.700 and 7.400, respectively. The median GRAVY value of structural proteins was −0.244, and the mean of WDPs was −0.232 (Figure 2b). The GRAVY value indicates the protein-water interactions, and a negative GRAVY value implies a hydrophilic protein [43]. GRAVY values of WDPs were found to be negative, and the average value was −1.175, aligning with WDPs’ hydrophilicity. II is one of the primary methods for predicting in vivo protein stability based on protein structures [44]. The higher the index, the more unstable the protein is. In this analysis, the median II value of structural proteins was 39.87 and the mean value of WDPs was 40.636, demonstrating that the structural proteins were more stable (Figure 2c). The calculations categorized almost all WDPs as unstable proteins, with an II of above 40, except for *XERO2*, whose II was −0.99. This consistent with previous studies that *XERO2*, a dehydrin, might rely on conserved K-segments motifs to interact with specific targets, different from other WDPs that may require folding to be functional [45]. Relatively, *XERO2* could even retain its disordered state at severe loss of water [46]. In terms of AI values, WDPs have lower values of AI compared with other structural proteins. Higher AI values are synonymous with higher thermostability [47], demonstrating that WDPs were less thermostable and poor in resistance of high temperatures (Figure 2d). Under a narrow range of temperature conditions, the WDPs were stable. Based on the above observations from theoretical studies, WDPs’ overall features were different from structural proteins.

IDPs are highly dynamic and contain hydrophilic residues such as charged and polar amino acids [48,49]. The FCR (fraction of charged residue) and NCPR (net charge per residue) values of IDPs contribute as determinants of corresponding conformation, leading to the classification of IDPs into five different classes [50]. We calculated FCR and NCPR values of these WDPs and adopted the functional classification scheme (Figure 2e, Table S3). Interestingly, the WDPs were distributed at roughly similar levels in these categories except for R4, which had only two proteins, and R5, which had no distribution. Figure 2e showed that 20 WDPs belonged to R1, which corresponds to proteins with globule or tadpole-like conformations [51]. Nineteen WDPs belonged to R3, where proteins tend to have coil-like, hairpin-like, or mixed conformations [52]. There were 15 WDPs in R2, with conformations likely to be mixed between R1 and R3 [51]. What determines IDPs conformation is chain-solvent, chain-chain, and solvent-solvent interactions, and the swollen coil prefers a good solvent [53,54,55]. Consequently, compact and roughly spherical WDPs promote favorable contacts with poor solvent, as the ions drawn into the core interact with the compact interior. These findings recapitulate the fact that flexible state-switching of WDPs will enhance their adaptability to the outer environment, highlighting their pivotal positions in the biological progress of all living cells.

### 2.3. Expression Profiles of the WDPs in Response to Abiotic Stresses

Adverse factors pose substantial challenges to geographic distributions and genetic diversity of plants as well as their morphological traits, and previous research manifests that IDPs are widely involved in the adaptation of plants to environmental stimuli [56]. The functional identification of WDPs in *Arabidopsis* require analysis of their expression in different tissues and response to various stresses. Therefore, we studied the expression levels of WDPs in shoots and roots under four different abiotic treatments. Since transcriptome data for 12 WDPs are not available, we herein focused on the remaining 44 WDPs.

**Figure 3:**
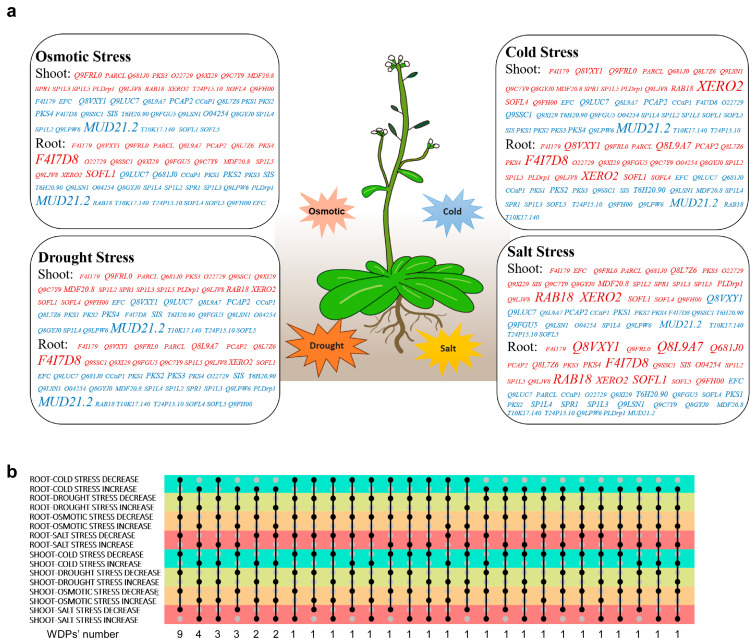
Expression patterns of the screened WDPs under abiotic treatment. (**a**). Collection of upregulated (red) and downregulated (blue) WDPs in shoots and roots under abiotic stresses. Log2 (foldchange) ≥ 0 was used as the threshold to determine the upregulation or downregulation. The larger the font, the larger the value of Log2 (foldchange). (**b**) Distribution of related stress-responsive WDPs in shoots and roots.

It was found that after exposure to osmotic stress, cold, drought, and salt treatment, about half of the WDPs showed increased expression, while the rest were observed to be inhibited. These were visualized in Figure 3a and Figure S2. Plants tend to adopt similar strategies to survive different stresses, such as stomatal closure in response to drought or salinity, and thus exhibit similar molecular mechanisms [57,58]. Certain WDPs displayed consistent expression patterns across different tissues, like the downregulation of *CCaP1, PKS1*, *PKS2*, *T10K17.140*, *Q9LPW6*, *T6H20.90*, *SP1L4*, *Q9LUC7,* and *MUD21.2* as well as the upregulation of *XERO2*, *Q9FRL0*, *Q9LJV8*, and *SP1L5* under all stress conditions (Figure 3a,b). As a dehydrin belonging to the highly hydrophilic LEA family ubiquitous in plants, *XERO2* was induced in response to stress, indicating its role as a positive regulator in the process [59]. The consistent expression trends of these WDPs suggested their crucial roles in plant growth and development. While some genes showed negligible expression, others like *RAB18* and *XERO2* may have partially overlapping influences on various adaptive processes to stimuli, as shown in Figure S2.

The varying expression levels of some WDPs indicated their complex and diverse patterns regulation. The expression of *SP1L2*, *SP1L5*, *PLDrp1*, *SOFL1,* and *SOFL4* in roots were induced under 4 °C, implying that these genes were regulated to participate in the chilling tolerance process, thus improving resistance (Figure S2a). However, the mRNA expression levels of some genes, like *Q9SSC1* and *MUD21.2*, gradually decreased with time extension. In the shoots, *MDF20.8*, *SPR1*, *SP1L5*, *PLDrp1,* and *RAB18* showed increased expression under cold stress (Figure S2a). Under drought stress, the expression of *PARCL, SP1L5*, *Q9C7Y9*, *Q9SSC1*, and *PCAP2* in roots exhibited increase (Figure S2b); that of *MUD21.2* decreased, conversely. It is possible that these proteins may be involved in essential processes like carbohydrate and energy metabolism to cope with specific drought conditions. In shoots after being treated for 24 h, *PARCL*, *PLDrp1*, *SP1L3*, *Q9SSC1*, and *RAB18* showed upregulation, while *Q8VXY1* and *MUD21.2* exhibited decrease (Figure S2b). Plants usually synthesize ABA to induce the closure of stoma and therefore prevent water loss by transpiration [60]. These facts imply some WDPs’ possible roles as transcription factors relative to the ABA signaling pathway, such as *PLDrp1*. In addition, some WDPs exhibited inconsistent fluctuation throughout the stress-triggered process, indicating that they may have unique functions. These WDPs may be expressed in response to specific stimuli as a result of specific conformational switchover to bind with target molecules.

### 2.4. Functional Prediction of WDPs

**Figure 4:**
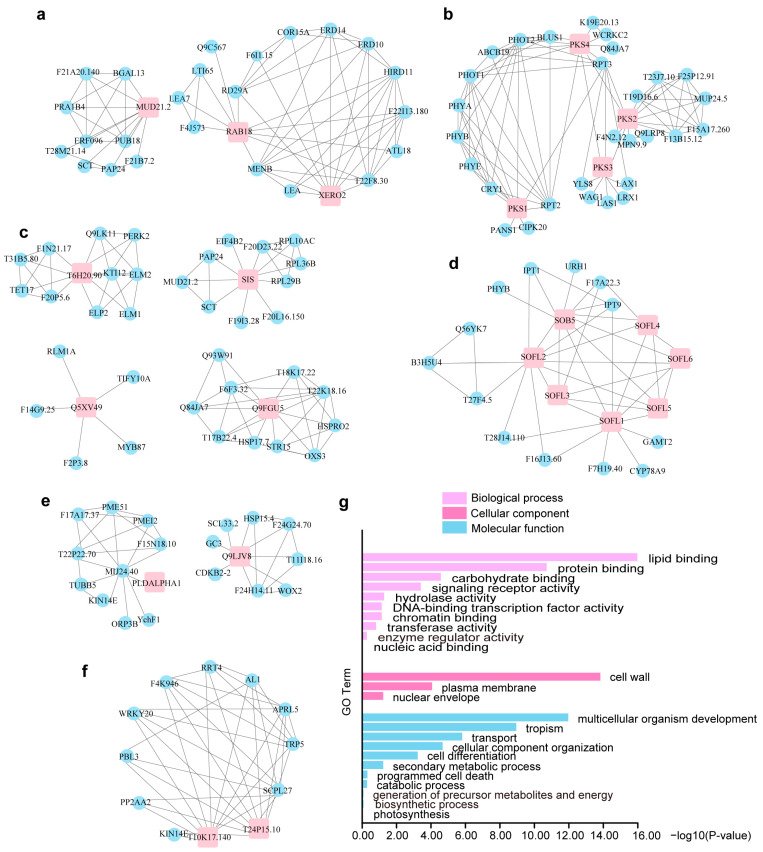
Protein-protein interaction networks of WDPs. (**a**) LEA family proteins. (**b**) PKS family proteins. (**c**) SIS-like family proteins. (**d**) SOB and SOB5-like family proteins. (**e**) *PLDAL–PHA1*. (**f**) Rho-GTPase activating gacO-like family proteins. (**g**) GO enrichment analysis of WDPs and their interacted proteins. In PPI, salmon squares represent WDPs, while blue circles represent proteins that interact with WDPs.

We further generated protein-protein interaction networks using the STRING database together with Cytoscape to decipher the physiological roles of these WDPs (Figure 4 and Figure S3). Lacking the relevant data, 18 of the screened WDPs had no corresponding statistics.

Gene Ontology (GO) functional enrichment analysis revealed that the rest 38 WDPs and their interacting proteins were mainly associated with multicellular organism development, tropism, transport cellular component organization, and cell differentiation et al. (Figure 4g, Table S4). Some WDPs had been functionally characterized and classified into specific classes. The representative PKS protein family, including *PKS1*, *PKS2*, *PKS3*, and *PKS4*, is involved in negatively regulating the signal transduction of photopigments and affect the developmental morphology of roots and leaves [61,62]. SIS interacted with members of the RPL family, which plays an important role in plant immunity and functions in the auxin pathway to optimize *Arabidopsis* growth and defense [63]. The interaction relationship between *SIS* and *MUD21.2* implied that *SIS* may act as a transcription factor to regulate the transcriptional process of genes related to stress tolerance [64,65]. Interestingly, *SIS* and *MUD21.2* shared similar expression patterns, except for the opposite expression profiles in response to salt treatment (Figure 3a). This observation may indicate their opposing functions in variable salinity.

The correlation between *CCaP1* and *NOP10* was noted. *NOP10* has emerged as a critical regulator in the modification of spliceosome small nuclear RNAs and stabilization of telomerase [66]. *CCaP1*, known as cytosolic Ca^2+^ binding protein in *Arabidopsis*, presented a common decrease faced with external stimulation (Figure 3a) [67]. Localized to the plasma membrane, *CCaP1* interacts with the plasma membrane H-ATPases *AHA1*/*AHA2* and functions as a regulator [68]. Taken together, the discovered *CCaP1* and *NOP10* interaction probably underlies the switchover mechanism related to stress response. *PARCL* showed upregulation under drought and osmotic stresses. Considering its colocalization with RNA in phase-separated condensates, *PARCL* probably forms liquid-liquid phase separations within cells in response to stimulus [69]. EFC might bind growth factors and regulate transcription, as its interacting proteins *F21P8.10*, *SAC2*, *HDG3* belong to proteins widely involved in chromatin function and epigenetic modifications [70]. *Q3E9A8* and *Q9XI29*, interacting with YDA, may act as a molecular switch to regulate the development of fertilized eggs in the MAPK pathway and regulate the development of stomata [71,72].

Overall, WDPs seem to be linked with a variety of cellular pathways enriched with transient interactions with diverse molecules, mostly participating in cell division and stress resistance. These may be common features of WDPs, as a result of the preference of specific amino acids and their plastic structures.

### 2.5. WDPs Tend to Undergo Liquid-Liquid Phase Separation

**Figure 5:**
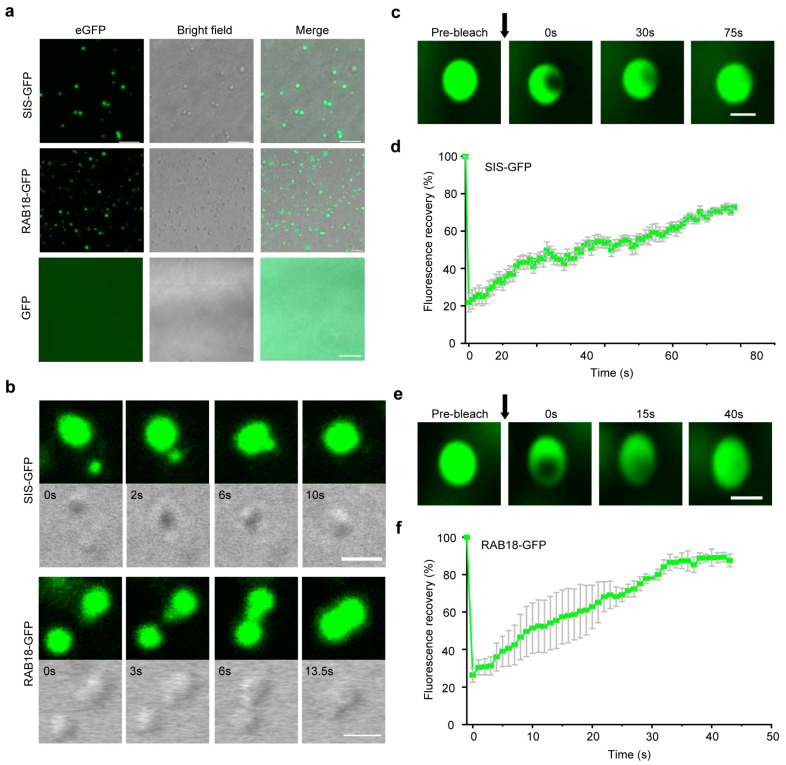
*SIS* of SIS-like family proteins and *RAB18* of LEA family proteins exhibit liquid-liquid phase separation characteristics in vitro. (**a**) *SIS–GFP* and *RAB18–GFP* form droplets in vitro. Scale bar, 5 μm. (**b**) Time-lapse microscopy showing fusion dynamics of *SIS–GFP* and *RAB–GFP* droplets. Time 0 indicates the time of start recording. Scale bar, 2 μm. (**c**,**e**) FRAP assay showing dynamic property of *SIS–GFP* or *RAB18–FP* droplets. The black arrow indicate time of photobleaching. Scale bar, 5 μm. (**d**,**f**). Quantification data of FRAP assays for *SIS–GFP* (**c**) or *RAB18–GFP* (**e**) droplets. Data are presented as mean ± SD (n = 10).

Previous studies have established that intrinsically disordered regions (IDRs) are a typically unique and common feature of eukaryotic proteins undergoing liquid-liquid phase separation (LLPS) [73]. In our study, we identified 56 WDPs, indicating that there is a high possibility of undergoing phase separation. Thus, we selected two proteins, one is *SIS* from SIS-like family proteins, another is *RAB18* from LEA family proteins, to test if they can undergo phase separation. We obtained proteins in *Escherichia coli* by optimizing the codons of *SIS* and *RAB18* for better expression and then connecting the proteins to the GFP tag (Figure S4). Proteins that undergo phase separation form droplets in an appropriate buffer solution [73]. Using laser confocal microscopy, we observed that two proteins formed smooth and round droplets in a buffer (25 mM Tris pH 7.4, 150 mM NaCl, 10% PEG), while GFP, used as a negative control, failed to form droplets under the same conditions (Figure 5a). Time-lapse microscopy also recorded that several small droplets merged into one large droplet in just over 10 s (Figure 5b, Videos S1 and S2). In addition, we used FRAP to monitor the molecular dynamics of *SIS–GFP* or *RAB18–GFP* within the droplets (Figure 5c,e). As demonstrated by the fluorescence redistribution after photobleaching (FRAP) assay, there was a robust molecular exchange between the bleached region and the surrounding area for both *SIS–GFP* and *RAB18–GFP* (Videos S3 and S4). Specifically, the recovery rate for *SIS–GFP* was 71.63% (Figure 5d), while that for *RAB18–GFP* was 89.34% (Figure 5f). Therefore, our results indicated that *SIS* and *RAB18* have the ability to form LLPS in vitro.

## 3. Discussion

In this study, we restricted the definition of WDPs with proteins of highly disordered degrees and obtained all WDPs in *Arabidopsis*. The applied criteria simultaneously considered impacts of the amino acid composition and structural information. The set of WDPs included known plant-specific PKS family, homologous proteins related to MAPK pathway, LEA and SOFL family responding to various abiotic stresses, and other homologous proteins. Our selection results are in accordance with previous and novel IDP researches, such as the inclusion of *RAB18*, *XERO2*, and *PARCL*. Belonging to the disordered dehydrin, *RAB18* and *XERO2* share the same repeat units and are strongly induced when exposed to cold [74]. In addition, SAXS experimental results also reveal a high degree of disorder in *PARCL* [69].

The physicochemical properties of WDPs were significantly different from those of structural proteins. They exhibited severe PI polarization, strong hydrophilic ability, and low fatty amino acid content. Based on FCR and NCPR values, most of the WDPs belonged to the R1 and R3 categories, with a small portion falling into the R2 region. Their conformations were mostly linear molten globule-like conformations or hairpin structures. These findings suggest that proteins with less compact conformation tend to carry a relatively large number of charges, resulting in the presence of hydrophobic residues and charge polarization [75]. Meanwhile, disorders affect proteins differently when interacting with others. These characteristics enable WDPs to bind other molecules through strong electrostatic forces, recruiting other biomolecules to achieve phase separation, and thus achieve functional diversification and instantaneous action. With a high content of polar amino acids, WDPs may bind more water under physiological conditions, aiding plants in surviving in their external environment. In addition to their role in resistance pathways, WDPs may be involved in various biological processes, such as acting as a microtubule binding protein, regulating the cytoskeleton, and cell division.

The evolution of species has been characterized by extensive gene loss [76]. As species complexity increased, so did WDPs. These WDPs have been preserved from early evolution and newly evolved with the increasing complexity of species, allowing them to adapt to environmental changes and achieve functional diversification. Such facts collectively explain why some WDPs have been preserved over the long course of evolution. Interestingly, some WDPs existed only in specific species, possibly resulting from plant differentiation from lower to higher complexity, and from simple to complex, as cells undergo different functional diversification.

The exact functions of WDPs have yet to be determined by experiments, but transcriptional and GO analysis indicated that these WDPs were widely involved in stress resistance and cell cycle regulation. Mechanisms discovered so far may be relevant for the WDPs to carry out their duties. A small number of proteins, like *F4I179* and *Q9LUC7*, lack relevant available data and demand further characterization. Due to their flexible conformation, studying how these WDPs function in biological processes presents significant challenges. Phase separation currently appears to be a primary structural form through which intrinsically disordered proteins exert their functions. Hnisz et al. [77] proposed a phase-separation model in mammals that explains features of transcriptional control, including the formation of super-enhancers, the sensitivity of super-enhancers to perturbation, and their transcriptional bursting patterns. Subsequently, it emerges that heterochromatin condensation in both animals and plants is orchestrated by phase separation, with the distinction lying in the involvement of species-specific proteins [78]. Moreover, proteins with intrinsically disordered regions, RNA-binding domains, and translation-related proteins dynamically forms stress granules (SGs) through LLPS in response to stress [79]. Recent research breakthroughs have uncovered that *Arabidopsis* De-Capping 5 (DCP5) detects hyperosmotic stress through molecular crowding and phase separation, forming DOSG stress granules that regulate the translatome and transcriptome to aid in plant osmotic stress adaptation [80]. Among the DOSG, there were RNA-binding proteins and mRNA, as well as translation initiation factors. In our study, WDPs were found to be responsive to a spectrum of stresses, including cold, salt, osmotic, and drought stress (Figure 3). By analyzing the protein-protein interaction network of *SIS*, we observed its associations with numerous ribosomal proteins, such as *F20D23.22*, *RPL36B*, *RPL29B*, *RPL10AC*, as well as the translation initiation factor *EIF4B2* (Figure 4c). In the case of *RAB18*, our investigation revealed significant interactions with various stress-related proteins and transcription factors, including *LTI65*, *HIRD11*, *ATL18*, *RD29A*, and *LEA7* (Figure 4a). Notably, we documented the in vitro phase separation of *SIS* and *RAB18* (Figure 5). It is reasonable to speculate that SIS or RAB18 may also form stress granules through liquid-liquid phase separation, recruiting a suite of transcription and translation machinery, after sensing external stress. Also, other WDPs in *Arabidopsis* are potential stress sensors and warrant further investigation.

## 4. Materials and Methods

### 4.1. Screening of Arabidopsis WDPs

We used the Advanced Search function of UniprotKB in Uniprot (https://www.uniprot.org/, Release 2022_05, Released on: Wed 14 December 2022) to database and set filtering parameters: (Proteome_id:UP000006548) AND (length:(xxx TO xxx)) AND (Region:Disordered:(xxx TO xxx)). Peptide sequences are composed of fewer than 50 amino acids each [81], so we commenced our investigation at amino acid number 0 and proceeded in increments of 50, setting a search interval of 50 amino acids. The length of unstructured area was established by setting the shortest to the longest length of intervals to guarantee the areas of disorder accounted for 90~100%. The retrieved results were then manually screened to ensure they were all consistent with the WDPs’ definition. Furthermore, any repeated sequences were removed using the Seqkit software (Version 2.2.0). The disordered protein sequences were subjected to comparative analysis with the *Arabidopsis* proteome, and the matched sequences underwent multiple sequence alignment using Uniprot-Align. The alignment results indicated the removal of specific short fragments. It should be noted that protein structures predicted by AlphaFold (https://alphafold.com, accessed on 13 February 2023) and MobiDB (https://mobidb.org, accessed on 13 February 2023) were also combined in order to ensure the totality of WDPs.

### 4.2. Classification Method of Arabidopsis WDPs

56 *Arabidopsis* WDPs were aligned to the *Arabidopsis* proteome and searched for homologous sequences of each WDPs (BLASTP, E-Value = 0.0001, Hits = 20). The results of homologous search and merge sequences were organized with the same or similar search results. Global multiple sequence alignment was performed on all sequences merged into one group to see if there were conservative motifs between the sequences, i.e., if the overall similarity of the sequences is high. If there were similar conservative regions, they were merged into one category based on sequence similarity.

### 4.3. Phylogenetic Analysis of Arabidopsis WDPs

The representative species of algae, mosses, monocotyledonous, and dicotyledonous plants were selected for phylogenetic analysis, including *Brassica oleracea*, *Nicotiana tabacum*, *Oryza sativa*, *Physcomitrium patens*, and *Chlamydomonas reinhardtii*. The proteome data of WDPs from Uniprot–Protome was employed by TBtools Blast in the search for local similarities and evolutionary relationships between *Arabidopsis* and the aforementioned species.

### 4.4. Physicochemical Properties Analysis of WDPs in Arabidopsis

A total of 56 structural proteins in *Arabidopsis* genome were randomly selected from Uniprot, and TBtools The Protein Paramter Calc (ProtParam-based) function was used to calculate the theoretical isoelectric point (PI), Instability index (II), Grand Average of Hydrophathicity (GRAVY) and Aliphatic Index (AI) of proteins. Unpaired *t*-test, Mann–Whitney test (unpaired, two-tail) statistical calculations, and plots were performed uniformly in Prism (GraphPad Prism 9.5.0 (730)).

### 4.5. Calculation of FCR and NCPR Values of WDPs

CIDER (https://pappulab.wustl.edu/CIDER/analysis/, accessed on 12 November 2023) was directly used to calculate the value of FCR and NCPR according to the protein sequences [82]. Positively charged amino acids include arginine (Arg, R) and lysine (Lys, K); negatively charged amino acids include aspartic acid (Asp, D) and glutamic acid (Glu, E). According to the calculation method provided by Rahul K Daset al. [83], N: total number of amino acid residues in the sequence; N+, N−: positively charged, negatively charged residual base; f+ = N+/N, f− = N−/N.

FCR = (f+ + f−); NCPR = |(f+ − f−)|

### 4.6. Analysis of Expression Patterns and Function Prediction of WDPs

The transcriptome expression data of *WDPs* was retrieved from TAIR *Arabidopsis* eFP Browser (https://www.arabidopsis.org/, accessed on 22 June 2023), selecting Abiotic Stress. The data was obtained from AtGenExpress, provided by Kilian using Affymetrix ATH1 microarray (generated at the RZPD, Berlin, Germany) [84]. We selected sample data from roots and shoots treated for 0 (control), 3, 6, 12, and 24 h, under cold treatment (4 °C), drought treatment (loss of 10% of fresh weight), salt treatment (150 mM NaCl), and osmotic stress treatment (300 mM D-Mannitol), respectively. Transcripts per million values for these WDPs were log2 transformed, and the heat map was constructed using TBtools (Version 0.665).

The interaction data pertaining to WDPs was extracted from STRING (https://cn.string-db.org/, accessed on 10 October 2023) and imported into Cytoscape_v3.9.1, to construct related interaction network and perform GO enrichment analysis.

### 4.7. In Vitro Phase Separation Assay

The full-length CDS sequences of *SIS* and *RAB18* were downloaded from the TAIR website. Then, the rare codons in the sequences were replaced with the commonly used codons in *E. coli*. Next, the bridge PCR method was used to fuse the target fragment with the GFP fragment. Subsequently, the fragment was ligated into a prokaryotic expression vector, and then the vector was transformed into the Transetta (DE3) strain of *E. coli*. The bacterial culture was grown to an OD_600_ of 0.5–0.6, IPTG was added to a final concentration of 1 mM, and the culture was induced at 20 °C, 180 rpm, for 16 h. After collecting the bacteria, the cells were lysed with lysis buffer (25 mM Tris, 150 mM NaCl, adjusted to pH 7.4 with HCl), and the protein was enriched by affinity chromatography, followed by elution with lysis buffer containing 200 mM imidazole. SIS–GFP and GFP protein was diluted to 10 μM, RAB18–GFP protein was diluted to 20 μM, in a buffer containing 25 mM Tris (pH 7.4), 150 mM NaCl, and 10% PEG as a crowding agent. Protein solution (5 μL) was loaded onto a glass-bottom cell culture dish and imaged using a laser scanning confocal microscopy (LSM880, ZEISS, Germany). The images presented are of droplets settled on the glass coverslip. For fluorescence recovery after photobleaching (FRAP) assay, GFP was excited at 488 nm and detected at 498–530 nm, and fluorescence intensity detection was performed for every second.

## 5. Conclusions

Here, we firstly characterize the meaning of WDPs and develop a set of standards for screening *Arabidopsis* WDPs at genome level. Thorough bioinformatics analysis we clarify the functional characterization of these WDPs. In vitro experiments revealed that these proteins have great potential to function in a phase-separation manner, providing clues for subsequent research on these proteins. Our results provide a valuable foundation for further investigation and functional analysis of WDPs, as well as for studies on the molecular mechanisms implementing various stages of plant development.

## Figures and Tables

**Table 1:** Classification of WDPs in *Arabidopsis.* White and gray backgrounds were used to separate different categories.

Protein ID	Gene ID	Gene Name	Description
F4I179	AT1G15840	/	Hypothetical protein
Q9M3G8	AT4G11430	*/*	Hydroxyproline-rich glycoprotein family protein
O82760	AT4G23110	*EFC*	Early flowering and curly leaves
Q8VXY1	AT5G28630	*/*	Glycine-rich protein
Q9FRL0	AT1G75190	*/*	Hypothetical protein
Q9LUC7	AT3G14670	*/*	Hypothetical protein
Q9C7W1	AT1G64370	*PARCL*	Phloem associated rna chaperone-like
Q8L9A7	AT4G27580	*/*	Phosphatidylinositol transfer SFH5-like protein
Q681J0	AT5G54095	*/*	Proteoglycan-like protein
Q9SCK5	AT3G49540	*T9C5.130*	Hypothetical protein
Q9LU05	AT5G44610	*PCAP2*	Plasma membrane-associated cation-binding protein 2
A0A1P8ASG6	AT1G04105	*/*	Hypothetical protein
Q9SXE9	AT1G62480	*CCaP1*	Vacuolar calcium-binding protein-like protein
Q8L7Z6	AT3G54680	*/*	Proteophosphoglycan-like protein
Q5XV49	AT5G05965	*/*	Cell wall RBR3-like protein
Q84WZ5	AT2G39855	*/*	Plant/protein
Q9SWI1	AT2G02950	*PKS1*	Protein phytochrome kinase substrate
Q9M9T4	AT1G14280	*PKS2*	
Q8GXS8	AT1G18810	*PKS3*	
Q9FYE2	AT5G04190	*PKS4*	
Q9XI29	AT1G15400	*MASS2*	MAPK substrates in the stomatal
Q9SSC1	AT1G80180	*MASS1*	
Q3E9A8	AT5G20100	*MASS3*	
Q9LZM9	AT5G02020	*SIS*	Salt induced serine rich
Q9STG0	AT3G46880	*T6H20.90*	Hypothetical protein
Q9FGU5	AT5G59080	*/*	Hypothetical protein
Q1G3N4	AT3G55646	*/*	Tprxl
Q9LSN1	AT3G17160	*/*	Hypothetical protein
Q9C7Y9	AT1G47970	*/*	Nucleolin
O04254	AT4G02140	*/*	Hypothetical protein
Q8GYJ0	AT4G22320	*BCL7A*	BCL-domain homolog
Q9FKA5	AT5G39570	*PLDrp1*	PLD regulated protein
Q9LJV8	AT3G29075	*/*	Glycine-rich protein
Q9FM74	AT5G55640	*MDF20.8*	Na-translocating NADH-quinone reductase subunit A
Q9LF22	AT5G15600	*SP1L4*	Protein SPIRAL1-like
B3H4F1	AT1G26355	*SP1L1*	
Q9LE54	AT1G69230	*SP1L2*	
Q9SJW3	AT2G03680	*SPR1*	
Q8LGD1	AT4G23496	*SP1L5*	
Q9S7P8	AT3G02180	*SP1L3*	
Q9FL02	AT5G66780	*MUD21.2*	Late embryogenesis abundant protein
P30185	AT5G66400	*RAB18*	Dehydrin protein family
P42758	AT3G50970	*Xero 2*	Dehydrin protein family
Q9M2Q5	AT3G57930	*T10K17.140*	Rho gtpase-activating gaco-like protein
O48526	AT2G42190	*T24P15.10*	
B6IDH8	AT1G58460	*SOFL6*	Hypothetical protein
Q67YG7	AT1G26210	*SOFL1*	Protein SOB FIVE-LIKE
Q9CA45	AT1G68870	*SOFL2*	Protein SOB FIVE-LIKE
F4J6N7	AT3G30580	*SOFL3*	Hypothetical protein
Q9FKQ9	AT5G38790	*SOFL4*	Hypothetical protein
Q8L9K4	AT4G33800	*SOFL5*	Hypothetical protein
Q9LEZ1	AT1G58460	*SOB5*	Hypothetical protein
Q9FH00	AT5G42290	*/*	Transcription activator-like protein
Q9LPW6	AT1G12830	*/*	Nucleolin
F4I7D8	AT1G11125	*/*	Hypothetical protein
O22729	AT1G61170	*/*	Hypothetical protein

## Data Availability

Data is contained within the article and Supplementary Material.

## Supplementary Materials

The following supporting information can be downloaded at: https://www.mdpi.com/article/10.3390/ijms26031117/s1.
