# Supplementary material for: Genome-Wide Characterization of Wholly Disordered Proteins in Arabidopsis
Source: Int J Mol Sci. 2025 Jan 28;26(3):1117. doi: 10.3390/ijms26031117 (PMC11817481; doi:10.3390/ijms26031117)
Supplement: Supplementary file 1 [file ijms-26-01117-s001.zip › ijms-3404484-supplementary.pdf]

Table S1. Evolutionary analysis of WDPs.

**Distribution**	**Proteins**
Specific in *Arabidopsis Thaliana*	F4I179, Q9M3G8, EFC, A0A1P8ASG6, CCaP1
Specific in Brassicaceae	Q8VXY1, Q9FRL0, Q9LUC7, PARCL, Q8L9A7,Q681J0, Q9SCK5, Q9LU05, SOFL2, SOB5
Specific in Dicotyledon	Q8L7Z6, Q9SWI1, Q9M9T4, Q8GXS8, F4I7D8, O22729, SIS, T6H20.90,Q9FGU5, Q5XV49, Q84WZ5, Q1G3N4, Q9LSN1, O04254, SOFL6
Specific in Angiospermae	Q9C7Y9, PKS4, Q9SSC1, Q3E9A8, Q9XI29, Q9LPW6, Q9LJV8,MUD21.2, T24P15.10, SOFL3, SOFL1, SOFL4, SOFL5
Angiosperm and Gymnospermae	Q8GYJ0, MDF20.8, SP1L4, SP1L1, SP1L2, SPR1 SP1L3, SP1L5, PLDrp1, RAB18 XERO2, T10K17.140, Q9FH00

Table S2. 56 randomly selected structural proteins in *Arabidopsis Thaliana*

Protein ID	Gene Name
A1A6M1	PTAC5
A2RVM0	TIC32
C0LGF4	FEI1
O04147	CPD
O04202	EIF3F
O04331	PHB3
O04905	KCY3
O22145	OSGP2
O22850	GPX3
O23403	PPD1
O23512	MES16
O48737	TRXM1
O49196	APK2
O49447	ADT3
O49627	ISU1
O64817	CSK23
O64903	NDK2
O80528	HASP
O80803	XTH17
O80920	PYL4
O81045	P24D8
O81417	SRPP
O82230	EBFC2
P10896	RCA
P35632	AP3
P42761	GSTFA
P43289	KSG3
P47924	RIBA1
P47998	CYSK1
P56761	PSBD
P92959	RK24
P93014	
Q02971	CADH7
Q2HIK4	PFD6
Q2V419	CKB12
Q38826	IAA8

Protein ID	Gene Name
Q38902	RAC1
Q39022	MPK2
Q39034	PER59
Q39112	GAOX3
Q39208	SC5D1
Q42565	ASB1
Q5EAF2	IP5PB
Q5XF78	GAMT2
Q67Y99	CLPF
Q6NME7	TEN1
Q6NQL6	LPPE2
Q6R0C4	MYB52
Q84VZ1	FHY1C
Q8GS60	HCAR
Q8GUN2	HINT1
Q8GWA2	MOC1
Q8GZ29	GH315
A1A6M1	PTAC5
A2RVM0	TIC32
C0LGF4	FEI1

Table S3. Classification of WDPs based on FCR and NCPR value.

R1: FCR<0.25&NCPR<0.25					
Protein	Length	Positively charged	Negatively charged	FCR	NCPR
F4I179	126	14	13	0.21	0.01
Q9M3G8	219	21	2	0.11	0.09
PARCL	178	25	17	0.24	0.04
Q8L7Z6	211	28	13	0.19	0.07
PKS2	442	56	50	0.24	0.01
PKS4	406	51	42	0.23	0.02
SIS	149	16	15	0.21	0.01
T6H20.90	153	17	13	0.20	0.03
Q9FGU5	135	15	14	0.21	0.01
Q5XV49	131	15	12	0.21	0.02
Q84WZ5	142	16	16	0.23	0.00
Q1G3N4	146	20	16	0.25	0.03
SP1L4	127	14	9	0.18	0.04
SP1L1	113	8	6	0.12	0.02
SP1L2	110	7	7	0.13	0.00
SPR1	119	9	8	0.14	0.01
SP1L3	122	10	6	0.13	0.03
SP1L5	99	10	10	0.20	0.00
RAB18	186	17	17	0.18	0.00
XERO2	193	23	17	0.21	0.03

R2: 0.25 ≤ FCR ≤ 0.35 & NCPR ≤ 0.35					
Protein	Length	positively charged	negatively charged	FCR	|NCPR|
O82760	148	29	21	0.34	0.05
Q9SCK5	166	17	39	0.34	0.13
A0A1P8ASG6	133	17	25	0.32	0.06
PKS1	439	61	50	0.25	0.03
PKS3	387	54	46	0.26	0.02
F4I7D8	265	37	38	0.28	0.00
O22729	251			0.28	0.01
Q9SSC1	138	27	13	0.29	0.10
Q3E9A8	113	19	11	0.27	0.07
Q9XI29	148	34	16	0.34	0.12
O04254	112	15	17	0.29	0.02
MDF20.8	149	20	26	0.31	0.04

MUD21.2	121	16	17	0.27	0.01
SOFL5	170	21	29	0.29	0.05
Q9FH00	110	13	23	0.33	0.09

R3: FCR > 0.35 & NCPR ≤ 0.35

Protein	Length	positively charged	negatively charged	FCR	|NCPR|
Q8VXY1	148	26	41	0.45	0.10
Q9FRL0	131	31	35	0.50	0.03
Q9LUC7	237	34	72	0.45	0.16
Q8L9A7	104	12	26	0.37	0.13
Q681J0	135	16	35	0.38	0.14
Q9LU05	168	32	43	0.45	0.07
Q9SXE9	152	14	49	0.41	0.23
Q9LSN1	165	15	77	0.56	0.38
Q9C7Y9	198	13	104	0.59	0.46
Q8GYJ0	238	31	59	0.38	0.12
Q9LPW6	213	19	77	0.45	0.27
PLDrp1	381	57	91	0.39	0.09
Q9LJV8	294	70	82	0.52	0.04
T10K17.140	141	34	32	0.47	0.01
T24P15.10	141	31	37	0.48	0.04
SOFL2	147	17	39	0.38	0.15
SOB5	144	12	43	0.38	0.22
SOFL3	128	26	28	0.42	0.02
SOFL1	148	19	34	0.36	0.10
SOFL6	148	19	34	0.36	0.10
SOFL4	131	24	28	0.40	0.03

R4: FCR > 0.35 & NCPR > 0.35

Protein	Length	positively charged	negatively charged	FCR	|NCPR|
Q9LSN1	165	15	77	0.56	0.38
Q9C7Y9	198	13	104	0.59	0.46

Table S4. Interaction and functional prediction of Arabidopsis WDPs

Gene family	Protein name	Predictive function	Interacting proteins	Related function
	EFC	Insulin-like growth factor binding protein	F21P8.10<br>SAC2<br>HDG3	BAH domain-containing protein; phosphatidylinositol-3,5-bisphosphate 5-phosphatase activity; transcription factor activity
	Q8VXY1		F20M17.3<br>F15D2.10	Response to ABA, tolerance to freezing
	PARCL	Colocalize with RNA in phase separation condensates and may be involved in RNA transport	F4J0E6<br>F19K23.23	Splicing factors of precursor mRNA; salt responds to endoplasmic reticulum stress pathway
	Q8L9A7	Involved in Ca^{2+} related signaling pathways	PCMPH87<br>M030	Involve in post-transcriptional processing of plant organelles mRNA
	Q681J0		MICU<br>Q8L9A7	Mitochondrial Ca^{2+} binding proteins; maintain low matrix calcium levels
	PCAP2	Respond to abiotic stress; microtubule binding protein; intracellular signal transduction	PCAP1<br>MAP70.1<br>SPR1	Microtubule binding protein; involve in plant cytoderm formation; involve in ABA-mediated stomatal closure
	CCaP1	Ca^{2+} binding proteins; respond to abiotic stress, like salt and light	NOP10	Formation of mRNA spliceosome；involve in the fusion of germ cells
PKS	PKS1 PKS2<br>PKS3<br>PKS4	Negatively regulate the signal transduction of photopigments, and regulate the morphology of roots and leaves;	PHOT<br>PHY	Plant phototropism and movement of chloroplast ; ubiquitination of proteasome

Gene family	Protein name	Predictive function	Interacting proteins	Related function
		respond to red/far red and blue light		
MAPK	Q9SSC1	Participants in the MAPK pathway; regulate stomatal development and closure; resist pathogenic microorganisms.	MPK<br>BASL	Response to oxidative stress; activation of transcriptional response to Agrobacterium stress; involved in asymmetric cell division
	Q3E9A8<br>Q9XI29		YDA	Functions in a MAPK cascade; regulate the development of stomatal cells
SIS-LIKE	SIS	Transcription factor to regulate genes responding to salt stress	RPL<br>MUD21.2	Component of the large subunit of the ribosome
	Q9STG0		TET17<br>ELM1-2<br>Q9MK11	Involved in cell division; Mitochondria; Cytochrome oxidation
	Q9FGU5	Respond to oxidative stress	T22K18.16<br>OXS3	Respond to oxidative\ozone stress
SPR1 and SPIRAL1	Q5XV49<br>SP1L1<br>SP1L2<br>SP1L3<br>SP1L4<br>SP1L5<br>SPR1	Maintain cell anisotropic growth; Involved in microtubule depolymerization induced by salt stress	TUBB6<br>MAP TOR<br>PCAP2	Involve in microtubule cytoskeleton organization and mitotic cell cycle; participate in the sensory processes of plant nutrition and hormone signals; participate in the adaptation process of stress; regulate plant growth and development
	Q9LPW6<br>PLDRP1	ABA signaling pathways; in		

Gene family	Protein name	Predictive function	Interacting proteins	Related function
		response to a variety of abiotic and biological stresses		
	Q9LJV8	May localize in the nucleus and involved in cell cycle regulation	CDKB2-2<br>WOX2	Regulate cell cycle and cell division; participate in asymmetrical division of plant zygotes
LEA	MUD21.2<br>RAB18<br>XERO2	Involve in plant resistance to adversity; respond to various abiotic stresses	COR15A<br>ERD1LEA<br>RD29A	
Rho-GTPase activating gacO-like protein	T10K17.140<br>T24P15.10	May be involved in immune function of plant	PBL3	Related to plant defense signals; respond to biological stress induced by SA and JA signaling
SOB snd SOB5-LIKE	SOB5<br>SOFL1<br>SOFL2<br>SOFL3<br>SOFL4<br>SOFL5<br>SOFL6	Involve in cytokinin and other plant hormone metabolism; regulate hormone levels	T28J14.110<br>F16J13.60	Microtubule-binding proteins; regulate cell division and participate in the development process mediated by cytokinin
	Q9FH00	transcriptional activator against biological pathogens or plant viruses	NAC091<br>WRKY61	Transcription factors that resist viral infection and participate in the basic viral resistance response pathway

Figure S1

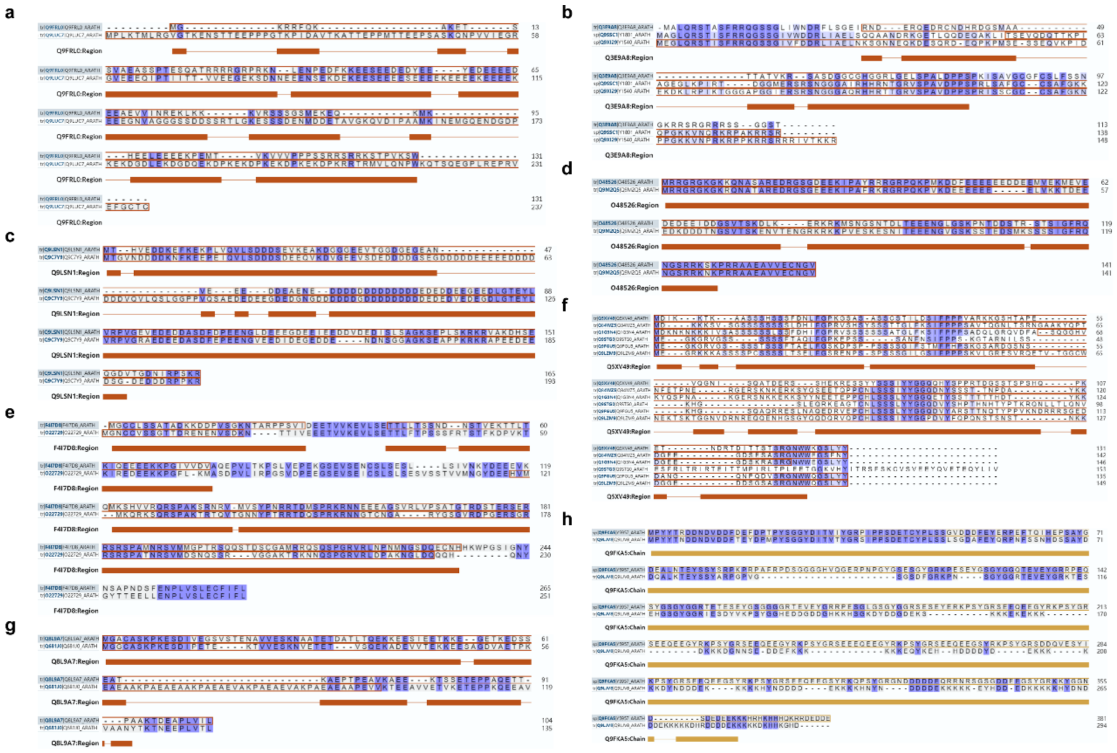

Figure S1. Multiple sequence alignment results. (a) Sequence comparison results of Q9FRL0H and Q9LUC7. (b) Sequence comparison results of Q9SSC1, Q3E9A8 and Q9XI29. (c) Sequence comparison results of Q9LSN1 and Q9C7Y9. (d) Sequence comparison results of T10K17.140(Q9M2Q5) and T24P15.10(O48526) (e) Sequence comparison results of F4I7D8H and O22729Q. (f) Sequence comparison results of SIS, T6H20.90, Q9FGU5, Q5XV49, Q84WZ5 and Q1G3N4. (g) Sequence comparison results of 8L9A7 and Q681J07. (h)PLDrp1 and Q9LJV8 sequence comparison results.

Figure S2

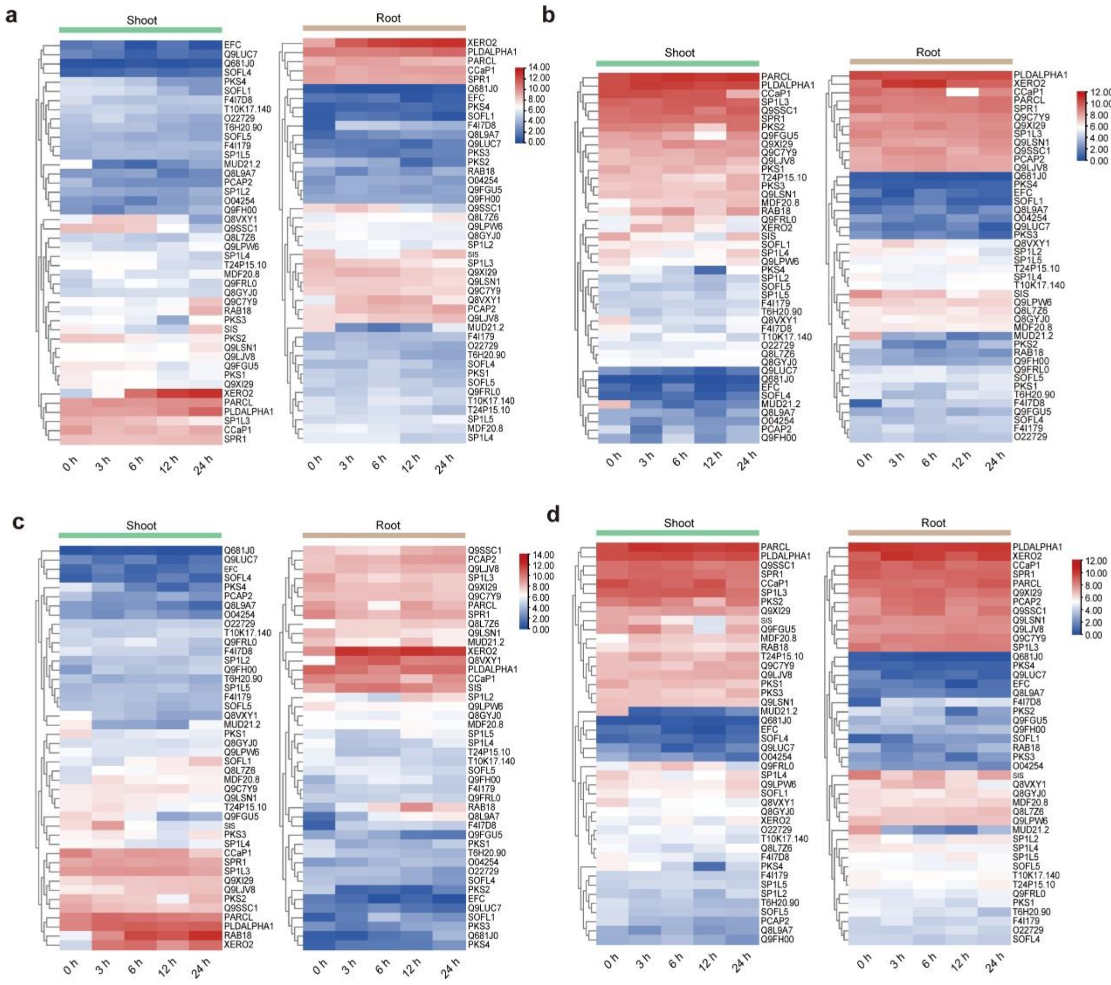

Figure S2. Clustering approach-based heatmap of the WDPs in response to various abiotic stresses, except for eight proteins without related data. (a). Expression of the WDPs in shoots and roots under cold. (b). Expression of the WDPs in shoots and roots under drought. (c). Expression of the WDPs in shoots and roots under salt. (d). Expression of the WDPs in shoots and roots under osmotic stress. The color gradient from blue to red indicates low to high expression. Transcripts per million values for these WDPs were transformed by log2(Fold change) and the heatmap was constructed using TBtools. Related treatments last for 3h, 6h, 12h, and 24 h.

Figure S3

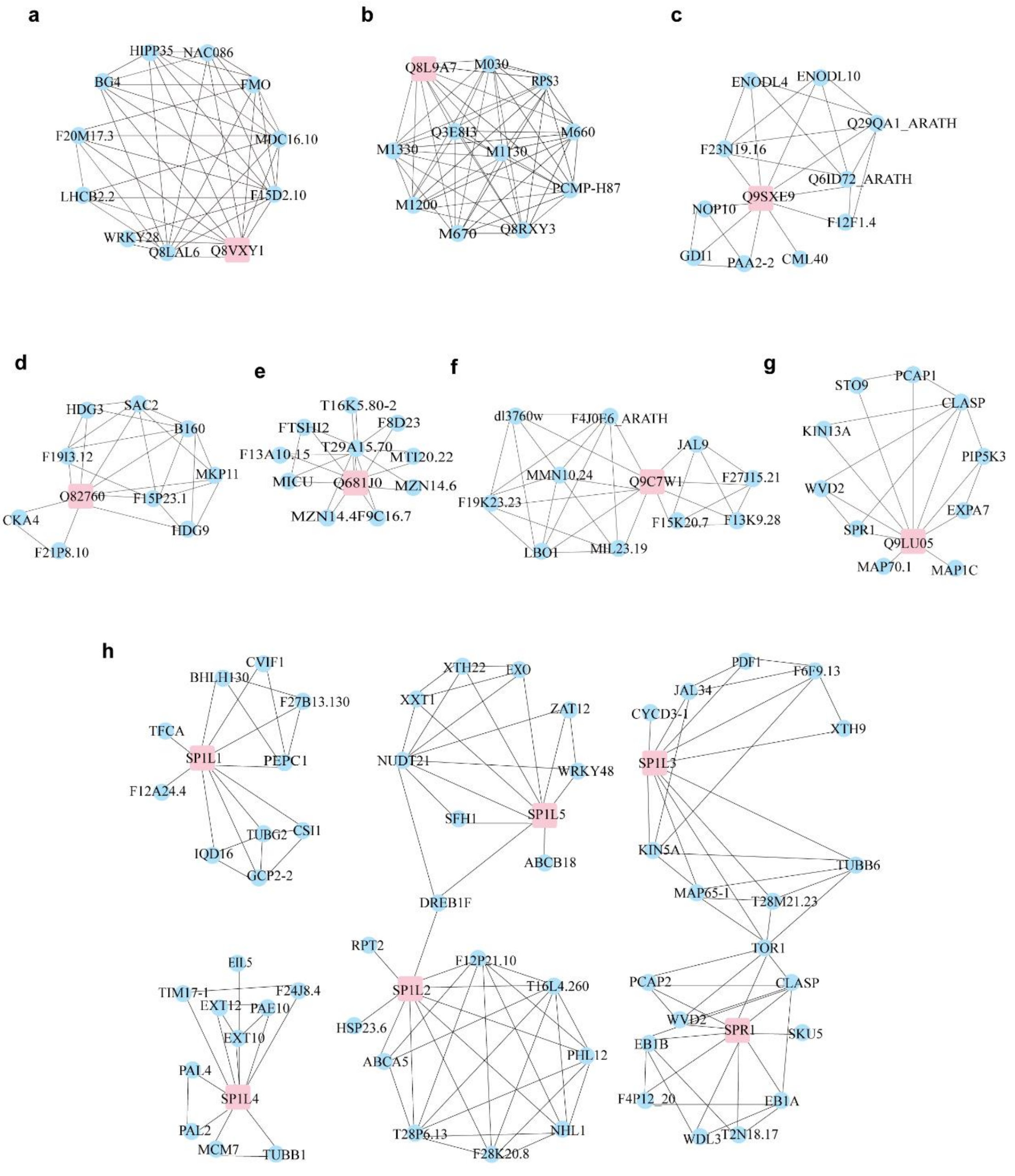

Figure S3. Protein-protein interaction networks of WDPs in *Arabidopsis thalianna*. (a). Q8VXY1. (b). Q8L9A7. (c). Q9SXE9. (d). O82760. (e). Q681J0. (f). Q9C7W1. (g). Q9LU05. (h). SPR1 and SPIRAL1 family proteins.

Figure S4

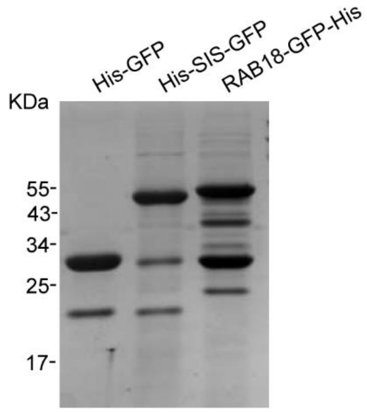

Figure S4. Protein purified from *Escherichia coli*.
